# Tachyarrhythmia in Wolff-Parkinson-White Syndrome

**DOI:** 10.5811/westjem.2016.4.30323

**Published:** 2016-06-16

**Authors:** Kelly Kesler, Shadi Lahham

**Affiliations:** University of California, Irvine, Department of Emergency Medicine, Irvine, California

## CASE PRESENTATION

**Figure 1:**
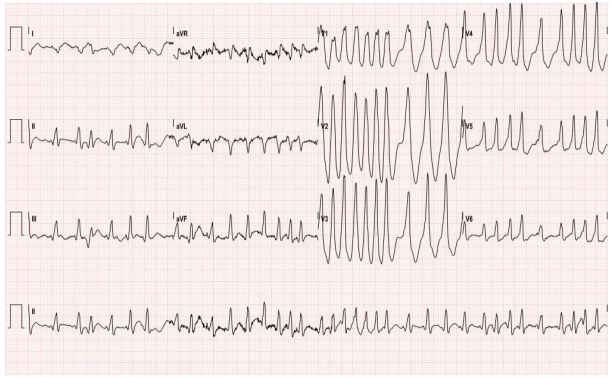
ECG showing tachyarrhythmia on initial presentation.

**Figure 2:**
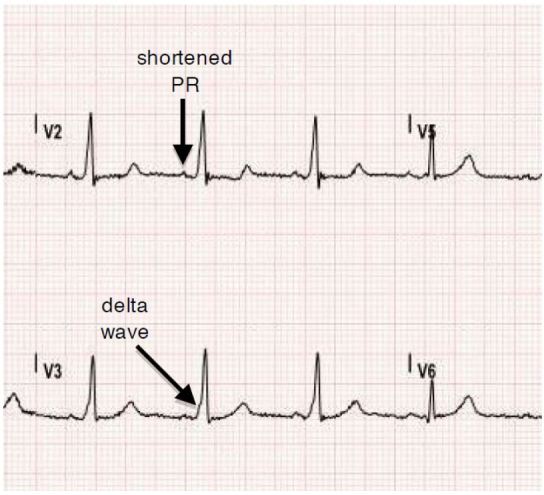
ECG after patient spontaneously converted to normal sinus rhythm. Delta waves and shortened PR interval appreciated.

A 29-year-old female with no significant past medical history presented with palpitations, nausea, diaphoresis and lightheadedness. Symptoms began 15 minutes prior to arrival. She reported several similar episodes previously that self-resolved within seconds, but had no previous medical evaluations for these symptoms. Initial vital signs were significant for blood pressure of 93/61, irregular heart rate between 180 and 200, respiratory rate of 18, and oxygen saturation of 99% on room air. Physical examination was otherwise unremarkable. The electrocardiogram (ECG) is shown in Figure 1. This was interpreted as atrial fibrillation with rapid ventricular rate, and the patient was treated with rate control with no effect. The patient later spontaneously converted to normal sinus rhythm and repeat ECG was notable for delta waves concerning for Wolff-Parkinson-White Syndrome (WPW) as seen in Figure 2. She was admitted to cardiology for cardiac ablation.

## DIAGNOSIS

Wolff-Parkinson-White (WPW) syndrome is a conduction disorder of the heart caused by pre-excitation accessory pathway resulting in tachyarrhythmias. The prevalence is approximately 0.07% of the population, and many patients often present with the chief complaint of “palpitations.”^1^ A diagnosis of WPW is made by certain characteristics identified on an ECG. These characteristics include a short PR interval < 0.12 seconds caused by faster electrical conduction through the accessory pathway than the atrioventricular (AV) node, and a delta wave, or upsloping of the QRS (Figure 2), due to rapid ventricular depolarization caused by the rapid conduction through the accessory pathway.^2^, ^3^ Diagnosing this disorder can be challenging, specifically when patients present with tachyarrhythmias and the pathognomonic delta wave becomes buried. The inherent rate of the AV node is approximately 180–200. Therefore, when a patient presents in an arrhythmia with a rate upwards of this intrinsic rate, an orthodromic atrioventricular reentrant tachycardia (AVRT) with a re-entrant component, such as WPW, should be immediately suspected 3.

**Figure 3:**
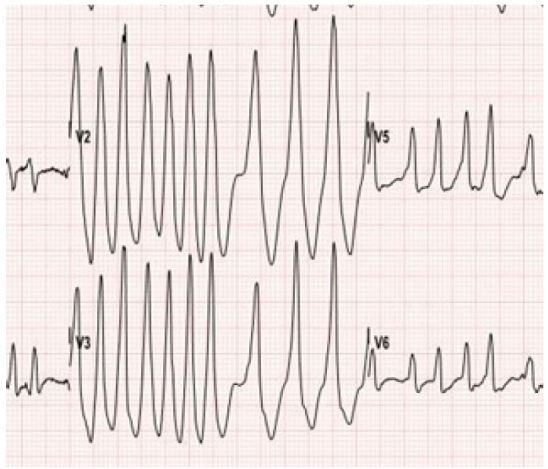
Rate of up to 300 on initial ECG.

In our case, the patient demonstrated a heart rate of up to 300 on the ECG (Figure 3). Once this patient converted back to normal sinus rhythm, the classic delta wave and short PR interval was easily identifiable (Figure 2). According to the 2014 American Heart Association guidelines for management of patients with atrial fibrillation, the class I recommendation regarding management of patients with pre-excited atrial fibrillation with rapid ventricular response includes IV infusion of procainamide if patient is hemodynamically stable, immediate synchronized cardioversion if the patient is unstable, and subsequent catheter ablation of the accessory pathway.^4^, ^5^ Administration of amiodarone, adenosine, beta blockers, and calcium channel blockers should be avoided as these will isolate the accessory pathway and thus predispose to fatal arrhythmias such as ventricular fibrillation by increasing the ventricular rate.^3^, ^4^, ^5^, ^6^, ^7^
